# Examination of exercise benefit/barrier perceptions of individuals with diabetes and affecting factors

**DOI:** 10.4314/ahs.v22i3.29

**Published:** 2022-09

**Authors:** Fatma Ersin, Derya Tülüce, Fatih Enzin

**Affiliations:** 1 Harran University, Faculty of Health Sciences, Public Health Nursing Department; 2 Harran University, Faculty of Health Sciences, Department of Internal Medicine Nursing; 3 Harran University, Faculty of Health Sciences, Department of Physical Therapy and Rehabilitation

**Keywords:** Individuals with diabetes, exercise benefit/barrier

## Abstract

**Background:**

Exercise, which is one of the health promotion behaviors, is extremely important in healthy life. This study was conducted to examine exercise benefit/barrier perceptions of individuals with diabetes and influencing factors.

**Method:**

This descriptive study was conducted in the Endocrine Polyclinics of a University Hospital with 285 individuals with Type 2 Diabetes between January and June 2020.

**Results:**

In this study, the average score of the exercise benefits subscale was 61.69 +14.79, the barriers subscale was 35.83 + 5.99, and the total score of the exercise benefits/barriers scale was 99.79 + 12.58. The total self-efficacy scale score was reported to be 59.74 + 9.46. A significant relationship was reported between the total mean score of the exercise benefits/barriers scale and having the opportunity to exercise, exercising regularly, and having a disease that prevents exercising. A significant difference was reported between the total mean score of the self-efficacy scale and the regular exercise status.

**Conclusion:**

Because of this study, the number of individuals who regularly exercised is insufficient, the mean exercise benefits/barriers scale score is not at the desired level, and exercise benefit/barrier perceptions are positively affected by the self-efficacy level.

## Introduction

Health promotion is defined as a process that helps individuals change their lifestyles to achieve the highest level of health. Positive health behaviors require to be acquired and maintained to protect and improve health. Exercise, which is one of the health promotion behaviors, is extremely important in healthy life^1^. Exercise is effective in preventing many chronic diseases^2^. When the risk factors of diabetes, which is one of the chronic diseases, are examined, combating physical inactivity has a critical importance in controlling the disease, treatment and management of risk factors^3^.

Exercise behaviors of individuals are closely related to their perception of benefits and barriers related to that behavior. A high perception of benefits and a low perception of barriers of the individual increase the possibility of performing the behavior^4^. Therefore, it is extremely important for individuals diagnosed with diabetes to be aware of the benefit and barrier perceptions of exercising. In a study using the health belief model scale, individuals with diabetes who exercise have higher benefit, lower barrier perceptions than those who do not exercise^5^.

Exercise behaviors of individuals are affected by multiple factors and one of the most important determinants is the self-efficacy level, which plays an important role in initiating behavior change and maintaining the behavior^4^. People with high levels of self-efficacy participate more in exercise programs adequately and regularly to reach a certain point in their behavior^2^,^6^,^7^. In another study, age, sex, and physical activity level affect the benefit and barrier perceptions of people with chronic diseases to a limited extent^8^.

There are exercise-related studies in individuals with diabetes^9^–^14^. A limited number of studies examining individuals' exercise benefit/barrier perceptions and their self-efficacy perceptions have been reviewed^15^. However, there are no studies investigating the exercise benefit/barrier perceptions and self-efficacy of individuals with diabetes. As a result, in protecting and improving the health of individuals with diabetes and keeping the disease under control, it is important to determine exercise benefit/barrier perceptions and self-efficacy of individuals. The results obtained will be effective in planning additional interventional studies to be performed. Therefore, this study was conducted to examine the exercise benefit/barrier perceptions of individuals with diabetes and influencing factors.

## Materials and Methods

The study was carried out at the Endocrine Polyclinics of a University Hospital between January and June 2020. It is a descriptive study.

### Population Sample

The population of the study comprised individuals with diabetes who applied to the Endocrine Polyclinics between January and June 2020. The sample comprised 285 diabetic individuals with a diagnosis of type 2 diabetes, at least primary school graduate and over 18 years of age, who voluntarily agreed to participate in the study.

### Data Collection Tools

The data were collected by the researchers using a face-to-face interview method. An introductory information form, the Exercise Benefits/Barriers Scale and the Self-Efficacy Scale were used to collect data.

Introductory Information Form: The introductory information form was prepared by researchers in line with the literature. The form comprised 16 questions concerning socio-demographic characteristics and exercise-related characteristics of participants^2^,^13^,^14^.

Exercise Benefits/Barriers Scale: The Exercise Benefits/Barriers Scale was developed by Sechrist, Walker and Pender (1987) to determine exercise benefit and barrier perceptions of individuals who will participate in exercise^16^. The validity and reliability of this scale in Turkey was conducted by Ortabağ, Ceylan, Akyüz and Bebiş (2010)^17^. The scale comprises a total of 43 items. The scale has four responses from four (strongly agree) to one (strongly disagree) on a forced Likert-type scale. The scale has two subscales: exercise barriers scale and exercise benefits scale. Each subgroup can be independently used. The barriers scale items are 4, 6, 9, 12, 14, 16, 19, 21, 24, 28, 33, 37, 40 and 42; the benefits scale items are items are 1, 2, 3, 5, 7, 8, 10, 11, 13, 15, 17, 18, 20, 22, 23, 25, 26, 27, 29, 30, 31, 32, 34, 35, 36, 38, 39, 41 and 43. The lowest score that can be obtained from the scale is 43 and the highest score is 172. The score range of the benefits scale is between 29 and 116, and the score range of the barriers scale is between 14 and 56. The sum of all items in the scale gives the total Exercise Benefits/Barrier scale score. The higher the total scale score, the more the individual understands the benefits of exercise. It is thought that the higher the score on the benefit scale, the higher the perceived benefit of the individuals. The higher the score on the disability scale, the higher the perceived disability of individuals. The Cronbach's alpha coefficient of the scale was determined to be 0.9517. In this study, the Cronbach's alpha coefficient of the scale was reported to be 0.87.

Self-Efficacy Scale: The scale is a self-assessment scale developed by Sherer and Maddux (1982) to evaluate behaviors and behavioral changes^18^. The validity and reliability of the Turkish form was made by Gözüm and Aksayan^19^. The scale comprises 23 items. For each item on a five-point Likert-type scale, the participants are asked to select one of the options: “it does not define me at all” (1), “it defines me a little” (2), “I am indecisive” (3), “it defines me well” (4), and “it defines me very well” (5). The scale consists of four subscales: initating a behavior subscale, maintaining a behavior subscale, completing a behavior subscale, persistence in the face of obstacle subscale. A minimum of 23 points and a maximum of 115 points can be obtained from the scale. A high total score obtained from the scale indicates that the individual's self-efficacy perception is at a good level. The Cronbach's alpha internal consistency coefficient, which includes all the statements of the scale, was 0.81, and the test-retest reliability was 0.92. In this study, the Cronbach's alpha coefficient of the scale was reported to be 0.65.

### Research Variables

The mean exercise benefits/barriers scale score was the dependent variables of the scale. The individuals' self-efficacy perception levels, age, sex, marital status, employment status, habits, social insurance status, economic status perception, family structure, exercise status, frequency of exercise, duration of exercise, presence of environment to exercise, and presence of a barrier to exercise were the independent variables of the scale.

### Data Analysis

The data were evaluated in the SPSS 17.0 package program. Number, percentage, mean, standard deviation, and maximum-minimum values were used in evaluating descriptive data. Whether the data showed normal distribution was determined by the Kolmogorov-Smirnov test. Because the data did not conform to normal distribution, the Mann-Whitney U test and the Kruskal-Wallis analysis were used in the analysis of the data. Because the mean scores of exercise benefits/barriers scale and self-efficacy scale were not normally distributed, the Spearman's correlation analysis was performed in the correlation between scales. Moreover, 0.05 was used as the significance level.

### Ethical Permissions

Before starting the collection of research data, written permissions were obtained from the relevant Clinical Research Ethics Committee (decision dated 27.01.2020 and numbered HRU / 20.02.27). Then, the permissions of the diabetic individuals included in the study were obtained, and all the data in the study were obtained according to the patients' statements.

## Results

The mean age of the participants was 51.47 ± 12.7, 36.8% of the participants were primary school graduate, 82%.5 were married, 66.3% were unemployed and 82.8% did not have social insurance. Moreover, 62.1% of the participants stated that their economic status was moderate, 79.3% lived mostly in the city, and 55.8% had a nuclear family. Note that 37.5% of the participants used cigarettes and 7.4% used alcohol (Table 1). The average year of diagnosis of the participants was 9.23 ± 4.73.

**Table 1:** Participants' socia-demografic characteristics

Characteristics	X ± SD	
**Age**	51.47 ± 12.78	
	**n**	**%**
**Education status** Primary school Middle School High school University and above	105 47 53 80	36.8 16.5 18.6 28.1
**Marital status** Married Single	235 50	82.5 17.5
**Employment status** Employed Unemployed	96 189	33.7 66.3
**Social insurance** Yes No	236 49	82.8 17.2
**Economic status** Poor Middle Good	44 177 64	15.4 62.1 22.5
**Longest living place** Villige City	59 226	20.7 79.3
**Family structure** Nuclear family Extend family Fragmented family	159 117 9	55.8 41.1 3.1
**Total**	**285**	**100.0**

When the exercise characteristics of participants were examined, 62.8% had the opportunity to exercise, 20.7% regularly exercised, and 39% of those who regularly exercised every day of the week and 27.1% exercised 75 -150 min a week, 80.4% did not have a barrier to exercise (Table 2).

**Table 2:** Participants' exercise characteristics

Characteristic	n	%
**Exercise opportunity** (n=285) Yes No	179 106	62.8 37.7
**Regularly exercise** (n=285) Yes No	59 226	20.7 79.3
**Exercise freguency (n=59)** Everday of the week 1–2 time in week 3–4 time in week 5 time in week	23 20 13 3	39.0 33.9 22.0 5.1
**Exercise duratin in week (n=59)** 45 minutes in week 60 minutes in week 75–150 minutes in week Other	8 17 16 18	13.6 28.8 27.1 30.5
**Have a disease barrier to** **exercise**(n=285) Yes No	56 229	19.6 80.4

In the study, the mean total exercise benefits/barriers scale score was 99.79 + 12.58, the mean exercise benefits subscale score was 61.69 + 14.79, and the mean exercise barriers scale score was 35.83 + 5.99. In terms of self-efficacy scale scores, the mean “initiating a behavior change” subscale score was 18.15 + 6.34, the mean “maintaining a behavior” subscale score was 16.15 + 5.24, the mean “completing a behavior” subscale score was 17.06 + 4.34 and the mean “persistence in the face of obstacles” subscale score was 8.38 + 2.45 and the mean total self-efficacy scale score was 59.74 + 9.46 (Table 3).

**Table 3:** Mean scores of the total and subscale of the Exercise benefit/barriers scale and Self Efficay Scale

Scales	X+SD (min-max)
**Exercise benefit/barriers scale**	
Exercise benefit subscale (EBS)	61.69±14.79 (29–101)
Exercise barriers subscale (EBS^+^)	35.83±5.99 (20–51)
Exercise benefit/barriers scale total (EBBS)	99.79±12.58 (60–139)
**Self Efficay Scale**	
Initating a behavior subscale (IB)	18.15±6.34 (8–39)
Maintaining a behavior subscale (MB)	16.15±5.24 (7–30)
Completing a behavior subscale (CB)	17.06±4.34 (5–25)
Persistence in the face of obstacle subscale (PFO)	8.38±2.45 (3–15)
Self efficacy scale total (SES)	59.74±9.46 (25–84)

There was a negative, moderately significant correlation between the mean exercise benefits subscale score and the mean exercise barriers subscale score (r = -. 480, p=.000) and there was a strong positive correlation between the mean exercise benefits subscale score and the mean total benefits/barriers scale score (r = .870, p = .000). There was a positive, very weak, significant correlation between the mean exercise benefits/barriers scale score and the mean “initiating a behavior change” subscale score of the self-effic and there was a positive, weak, significant correlation between the mean exercise benefits/barriers scale score and the mean “maintaining a behavior” subscale score (r = .280, p = .000). There was a negative, weak, significant correlation between the mean exercise benefits/barriers scale score and the mean “completing a behavior” subscale score of the self-efficacy scale (r = -. 200, p = .001) and there was a negative, very weak, significant correlation between the mean exercise benefits/barriers scale score and the mean “persistence in the face of obstacles” subscale score (r = -. 197, p = .001). There was a positive, very weak, significant correlation between the mean total exercise benefits/barriers scale score and the mean total self-efficacy scale score (r = .156, p = .000) (Tablo 4)

**Table 4:** Correlation between the mean total exercise benefits/barriers scale score and subscale scores and the mean total self-efficacy scale score and subscale scores

Scales and subscale	EBS	EBS+	EBBS	IB	MB	CB	PFO	SES
EBS	1							
EBS^+^	-.480 **.000**	1						
EBBS	.870 **.000**	-.065 .273	1					
IB	.312 **.000**	-.299 **.000**	.187 **.002**	1				
MB	.385 **.000**	-.260 **.000**	.280 **.000**	.756 **.000**	1			
CB	-.319 **.000**	.296 **.000**	-.200 .001	-.484 **.000**	-.458 **.000**	1		
PFO	-.227 **.000**	.149 **.012**	-.197 **.001**	-.302 **.000**	-.222 **.000**	.488 **.000**	1	
SES	.226 **.000**	-.140 **.004**	.156 **.008**	.770 **.000**	.770 **.000**	-.012 .843	.135 **.022**	1

E BS: Exercise benefit subscale; EBS+: Exercise barriers subscale; EBBS: Exercise benefit/barriers scale; IB: Initating a behavior; MB: Maintaining a behavior; CB: Completing a behavior; PFO: Persistence in the face of obstacle; SES: Self efficacy scale

A statistically significant difference was reported between the mean exercise benefits/barriers scale score and education status (KW = 22.014, p = .000), employment status (U = 6762.50, p = .000), social insurance status (U = 4645.00, p = .030), and family structure (KW = 14.628, p = .001) (Table 5).

**Table 5:** Distribution of the mean exercise benefits/barriers scale score according to the socio-demographic characteristics of the participants

	EBS	EBS^+^	EBBS	IB	MB	CB	PFO
**Education status** Primary school Middle School High school University and above	65.44±14.36 63.34±17.15 62.68±14.18 55.14±12.11	35.16±5.81 35.06±6.58 36.42±6.89 36.78±5.09	102.56±12.52 100.38±14.28 101.83±12.33 94.41±10.06	19.20±6.97 18.57±6.17 18.34±5.75 16.40±5.63	16.80±5.72 17.11±4.83 16.25±4.66 14.69±4.96	16.37±4.46 16.83±5.07 17.47±3.98 17.83±3.85	8.03±2.52 8.47±2.41 8.72±2.51 8.58±2.31
	K-W=26.321 **p=0.000**	K-W= 1.665 p=0.645	K-W=22.014 **p=0.000**	K-W=9.065 **p=0.028**	K-W=10.828 **p=0.013**	K-W=4.379 p=0.223	K-W=3.303 p=0.347
**Marital status** Married Single	61.39±14.95 63.08±14.08	35.72±6.12 36.34±5.34	99.42±12.68 101.48±12.07	18.00±6.23 18.84±6.86	16.08±5.21 16.50±5.45	17.06±4.49 17.08±3.57	8.53±2.49 7.68±2.11
	U=5490.50 p=0.467	U=5596.50 p=0.598	U=5450.50 p=0.422	U=5548.50 p=0.537	U=5643.50 p=0.661	U=5826.50 p=0.927	U=4801.50 p=0.041
**Employment status** Employed Unemployed	58.35±14.39 63.38±14.73	35.46±5.81 36.02±6.08	96.36±12.38 101.51±12.35	17.35±5.75 18.56±6.59	15.53±4.86 16.47±5.41	17.74±4.01 16.71±4.47	8.97±2.55 8.08±2.35
	U= 7265.50 **p=0.006**	U= 8456.00 p=0.348	U= 6762.50 **p=0.000**	U= 8229.00 p=0.199	U= 8299.50 p=0.239	U= 8005.00 p=0.104	U= 7292.50 **p=0.006**
**Social insurance** Yes No	60.71±15.08 66.37±12.41	36.14±5.96 34.37±5.98	99.12±12.59 102.94±12.14	18.35±6.53 17.18±5.29	16.31±5.24 15.39±5.22	17.14±4.39 16.65±4.09	8.39±2.46 8.31±2.39
	U= 4459.00 **p=0.012**	U= 4744.00 **p=0.048**	U=4645.00 **p=0.030**	U= 5310.00 p=0.368	U= 5195.00 p=0.262	U= 5274.00 p=0.332	U= 5667.50 p=0.826
**Economic status**							
Poor Middle Good	60.02±13.52 60.71±14.67 60.03±15.04	33.77±4.95 35.83±5.89 37.25±6.55	104.34±11.78 98.72±12.55 99.56±12.68	19.89±5.84 17.82±6.27 17.88±6.74	16.84±5.43 15.87±5.06 16.47±5.61	15.73±3.76 17.33±4.15 17.21±5.08	8.18±2.45 8.27±2.40 8.84±2.55
	KW=9.438 **p=0.009**	KW=7.891 **p=0.019**	KW=5.721 p=0.057	KW=4.900 p=0.086	KW=1.648 p=0.439	KW=5.828 p=0.054	KW=3.204 p=0.202
**Longest living** place Villige City	65.81±15.81 60.63±14.36	35.14±5.97 36.01±5.99	102.97±12.79 98.96±12.42	19.64±7.17 17.77±6.07	16.89±6.23 15.96±4.96	16.21±4.18 17.28±4.37	8.21±2.59 8.43±2.41
	U= 5445.50 **p=0.042**	U=6078.50 p=0.367	U= 5545.00 p=0.064	U= 5666.50 p=0.101	U= 6138.50 p=0.426	U= 5655.50 p=0.097	U= 6205.50 p=0.497
**Family structure** Immediate family Extend family Fragmented family	58.57±13.05 65.21±16.32 71.00±8.09	36.45±6.02 35.12±5.90 34.11±5.86	97.53±11.00 102.27±14.07 107.22±10.13	17.85±6.14 18.76±6.68 15.56±4.45	15.75±4.89 16.71±5.77 16.00±3.74	17.25±4.22 16.86±4.51 16.33±4.66	8.45±2.30 8.29±2.66 8.33±2.18
	KW= 18.222 **p=0.000**	KW=3.442 p=0.179	KW=14.628 **p=0.001**	KW=2.248 p=0.325	KW=1.734 p=0.420	KW=0.441 p=0.802	KW=0.238 p=0.888

EBS: Exercise benefit subscale; EBS+: Exercise barriers subscale; EBBS: Exercise benefit/barriers scale; IB: Initating a behavior; MB: Maintaining a behavior; CB: Completing a behavior; PFO: Persistence in the face of obstacle

There was a statistically significant correlation between the mean total exercise benefits/barriers scale score and the ability to exercise (U = 7140.00, p = 0.000), exercising regularly (U = 4802.00, p = 0.001) and having a disease that prevents exercise (U = 4732.00, p = 0.002) (Table 6).

**Table 6:** Distribution of the mean exercise benefits/barriers scale score according to the exercise-related characteristics of the participants

	EBS	EBS+	EBBS	IB	MB	CB	PFO
**Exercise opportunity**							
Yes No	57.86±13.94 68.15±13.96	37.36±5.60 33.25±5.74	97.64±12.47 104.39±11.97	17.41±6.36 19.39±6.13	15.69±5.25 16.92±5.16	17.66±4.22 16.04±4.38	8.54±2.61 8.11±2.13
	U= 5627.00 **p=0.000**	U=5672.00 **p=0.000**	U=7140.00 **p=0.000**	U=7465.50 **p=0.006**	U=7959.50 **p=0.023**	U=7505.50 **p=0.003**	U=8714.0p=0.246
**Regularly exercise**							
Yes No	53.95±11.42 63.71±14.92	38.63±5.93 35.10±65.79	95.19±9.99 100.98±12.92	15.53±6.14 18.84±6.22	14.31±4.99 16.64±5.21	17.98±4.55 16.82±4.27	8.71±2.54 8.29±2.42
	U=4019.50 **p=0.000**	U=4397.00 **p=0.000**	U=4802.00 **p=0.001**	U=4519.50 **p=0.000**	U=4810.50 **p=0.001**	U=5649.50 p=0.070	U=5964.0p=0.070
**Have a disease barrier to exercise**							
Yes No	69.14±14.78 59.86±14.24	34.04±5.64 36.27±5.99	104.89±12.13 98.53±12.39	20.04±6.09 17.69±6.32	17.29±5.26 15.88±5.21	15.21±4.36 17.51±4.23	7.43±2.21 8.62±2.45
	U=4258.50 **p=0.000**	U=5305.00 **p=0.045**	U=4732.00 **p=0.002**	U=4949.50 p=0.008	U=5350.00 p=0.054	U=4577.50 **p=0.001**	U=4540.0**p=0.001**

EBS: Exercise benefit subscale; EBS+: Exercise barriers subscale; EBBS: Exercise benefit/barriers scale; IB: Initating a behavior; MB: Maintaining a behavior; CB: Completing a behavior; PFO: Persistence in the face of obstacle

## Discussion

Because of the restrictions during the Covid-19 pandemic, the intensive use of technology has led to an increase in the number of physically inactive individuals and the maintenance of health-enhancing behaviors has been negatively affected. For this reason, health-enhancing behaviors are very important for individuals diagnosed with diabetes to keep their disease under control and to adapt to the treatment process. This study was performed to determine exercise benefits/barriers perceptions of diabetic individuals and the factors affecting them.

Because the studies conducted with the exercise benefits/barriers scale in diabetic individuals are limited, the results of this study were discussed with the results of the studies conducted in different sample groups.

Exercise is considered an important treatment parameter for diabetes mellitus^20^. Participants in our study were reported to regularly exercise at a low level (20.7%). Moreover, 27.1% of the participants who exercised regularly exercised at the desired level. In a study, researchers reported that despite the positive effects of exercise, only few patients with diabetes maintained physical activity and in those who exercised, the intensity of exercise was extremely low^10^. For the positive effects aimed in diabetic patients to occur, the recommended exercise prescriptions should comprise aerobic exercise workouts to be performed at least 3–7 days a week (two days in a row) in combination with resistance training and normal range of motion exercises to be performed 2–3 days a week^21^. In order for the exercise to be effective in patients with diabetes, it is important to do it at a sufficient intensity, frequency and awareness. The fact that the ratio of the participants who exercised in this study was not at the desired level can be explained by both the restrictions experienced during the pandemic and the insufficient level of awareness. Moreover, the mean exercise benefits subscale score of the exercise benefits/barriers scale may have affected regular execise status.

In this study, the mean exercise benefits/barriers scale score was 99.79 + 12.58. In a study conducted with nursing and medical students, the researchers reported a higher mean exercise benefits/barriers scale score than this study (122.98 + 15:47)^22^. Because the highest score that can be obtained from the exercise benefits/barriers scale is 172, the benefit perceptions of the participants in this study are not at a sufficient level.

Exercise benefit perception means subjective evaluation derived from exercise behavior^4^. In this study, the mean exercise benefits subscale score of the participants was reported to be 61.69 + 14.79. In a study conducted by Ransdell et al. with women in 2004, the mean exercise benefits subscale score of the participants was higher than our study (92.71 ± 8.30)^23^. In another study conducted with nursing students, the mean exercise benefits subscale score was reported to be 90.68 ± 12.9817. In the study conducted by Doğan and Ayaz with nurses, the mean exercise benefits subscale score was 89.3 ± 11.6 2. Because the highest score that can be obtained from the mean exercise benefits subscale score of the exercise benefits/barriers scale is 116, the mean score of the participants in the exercise benefits subscale in this study is low. Furthermore, this result suggests that the participants do not have sufficient awareness of exercise.

Perceived barrier to an action is related to the barriers encountered in doing that action. Perceived barriers may prevent starting a new activity or reduce commitment to continued activity^4^. In Doğan and Ayaz's study, perceived barriers are an important factor affecting exercise^2^. In this study, the mean exercise barriers subscale score of the exercise benefits/barriers scale was 38.12 + 6.51. The mean exercise barriers subscale score was 50.17 ± 4.79 in the study of Ransdell et al. and 28.66 ± 5.50 in the study of Ortabağ (2009)^17^,^23^. In another study conducted with nurses, the mean exercise barriers subscale score of the participants was 31.4 ± 5.4 2. Because the highest score that can be obtained from the mean exercise barriers subscae score of the exercise benefits/barriers scale is 56, the mean score of the participants in the exercise barriers subscale in this study is not at the desired level. The reason for this result suggests that individuals with diabetes do not have suitable conditions for exercise because of measures taken during the Covid-19 pandemic.

One of the important factors affecting developing health-enhancing behaviors is self-efficacy level. Self-efficacy expresses individuals' belief in performing a behavior^2^,^4^. In this study, the mean self-efficacy scale score was reported to be 59.55 + 9:45.

In the study of Doğan and Ayaz, the mean self-efficacy scale score of the nurses was reported to be 74.1 ± 12.0 2. In this study, the mean self-efficacy scale score of the participants is not at the desired level. Furthermore, a positive, very weak, significant correlation was reported between the mean self-efficacy scale score and the mean exercise benefits/barriers scale score of diabetic individuals (r = .157, p = 0.009). The obtained results indicate that the Covid-19 pandemic has lowered the participants' belief in performing health-protective and health-enhancing behaviors.

In this study, the education status, employment status, social insurance status, economic status, place of residence they lived the longest, family type, opportunity to exercise, regular exercise status, presence of a disease barrier to exercise, smoking status affected the mean exercise benefits subscale score of the exercise benefits/barriers scale of the participants. Furthermore, the economic status, place of residence they lived the longest, opportunity to exercise, regular exercise status, presence of a disease barrier to exercise, and smoking status affected the mean exercise barriers subscale score of the exercise benefits/barriers scale of the participants. In a study, it was reported that, unlike this study, marital status affects the mean exercise barriers subscale score 15.

In this study, the mean exercise benefits subscale score of the exercise benefits/barriers scale of the participants who were married was higher than single individuals; however, the mean exercise barriers subscale score of the exercise benefits/barriers scale of the participants who were married was lower than single individuals. Unlike the mentioned study, in the study conducted by Bakır and Hisar, the mean exercise barriers subscale score of the single individuals was lesser than that of the married participants 15. The results of the study demonstrate that married individuals are aware of the benefits and barriers of exercise behavior that are included in healthy lifestyle behaviors, which is an expected result.

In this study, it was determined that the mean exercise benefits subscale score of the exercise benefits/barriers scale of the participants who exercised regularly was low while the mean exercise barriers subscale score was high. In a study, the mean exercise benefits subscale score and barriers subscale score of nurses who exercised regularly were reported to be statistically significantly high 2. In a study conducted by Arısoyusing the health belief model scale, the benefit and barrier perceptions of individuals who exercised were reported to be higher than those who did not exercise 5. Regular exercise has an effect on glycemic results, weight loss and cardiovascular risk factors in individuals with diabetes 24. However, in a qualitative study, while it was emphasized that exercise is a source of motivation for a healthy life, patients experienced certain negative situations after exercise (experiencing hypoglycemia, increasing carbohydrate intake.). These results cause uncertainty in patients about benefits of exercise 25. The low-level exercise benefit perceptions of participants who regularly exercised in the mentioned study suggests that they may have faced different problems after exercise.

## Limitations of the study

During the Covid-19 pandemic, the restrictions on individuals with chronic diseases and the fear of individuals with diabetes getting infected caused a decrease in the number of diabetic individuals coming to the outpatient clinic. Therefore, the limited number of diabetic individuals reached is a limitation of the study.

## Conclusion and Recommendations

In the study, the number of individuals who regularly exercise is not sufficient. Therefore, it is recommended to conduct interventional studies that will enable individuals with diabetes to understand the importance of exercise in controlling their disease.

Moreover, considering the fact that the mean exercise benefits/barriers scale score is not at the desired level, and that the exercise benefit/barrier perception is affected by multiple factors, additional studies should be conducted to increase exercise benefit/barrier perception.

Furthermore, it was seen in the study that the exercise benefit/barrier perceptions of the participants were affected by their self-efficacy level. Therefore, training and exercise programs should be developed to identify, initiate and maintain behaviors that will increase the level of self-efficacy of individuals with diabetes and to identify barriers to these behaviors.
